# *QuickStats:* Age-Adjusted Death Rates*^,^^†^ for Pedestrians Involved in a Collision with a Motor Vehicle,^§^ by Race and Hispanic Origin^¶^ — National Vital Statistics System, United States, 2021

**DOI:** 10.15585/mmwr.mm7224a7

**Published:** 2023-06-16

**Figure:**
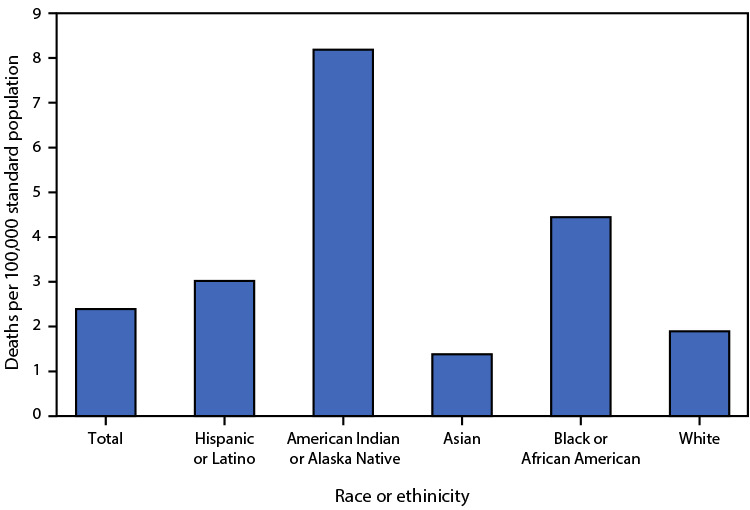
In 2021, a total of 8,392 deaths from pedestrian-involved collisions with motor vehicles occurred. The age-adjusted death rate from such collisions was highest for American Indian or Alaska Native persons (8.2 deaths per 100,000 standard population), followed by Black or African American (4.4), Hispanic or Latino (3.0), White (1.9), and Asian (1.4) persons.

**Source:** National Vital Statistics System, Underlying Cause of Death, 2018–2021. https://wonder.cdc.gov/ucd-icd10-expanded.html

